# Antidepressant Therapy in Severe Depression May Have Different Effects on Ego-Dystonic and Ego-Syntonic Suicidal Ideation

**DOI:** 10.1155/2011/896395

**Published:** 2011-05-12

**Authors:** Louise Brådvik, Mats Berglund

**Affiliations:** ^1^Division of Psychiatry, Department of Clinical Sciences Lund, Lund University Hospital, 221 85 Lund, Sweden; ^2^Department of Clinical Alcohol Research, University Hospital MAS, Lund University, Malmö, Sweden

## Abstract

The objective of the present study was to investigate whether ego-dystonic and ego-syntonic suicidal ideation occurred at different frequencies during antidepressant therapy. A blind evaluation has been performed on records of 100 suicides with a primary severe depression and 100 matched controls, admitted to the Department of Psychiatry, Lund, Sweden. Ego-dystonic suicidal ideation was more commonly reported during adequate treatment as compared to ego-syntonic ideation (*P* = .004). Men who committed suicide during adequate antidepressant therapy more often reported ego-dystonic suicidal ideation earlier in their lives compared with those who were not treated (*P* = .0377). This may indicate that treatment failure for ego-dystonic ideation was a precursor of their suicides. Consequently, ego-dystonic ideation seems to show a poorer response to antidepressant therapy as compared to ego-syntonic ideation, which may be more directly related to depression. Ego-dystonic ideation is proposed to be related to depressive psychosis.

## 1. Introduction

Suicidal ideation is a precursor of completed suicide, and it is important to evaluate the effect of treatment on its occurrence. Reduction of suicidal ideation has been shown during antidepressant medication [1–5], or at least in some individuals [6, 7], and after ECT [8–10]. However, an emergence of suicidal tendencies has also been found [11–13]. 

These studies were based on undifferentiated suicidal ideation. Previous research by Ringel on suicidal ideation has distinguished between active and passive suicidal ideation, depending on whether the individual keeps some subjectively active position or is passively overwhelmed by suicidal thoughts [14]. In addition, Akiskal has described that some depressed people could be tormented with suicidal obsessions and constantly resist unwanted suicidal urges or impulses to destroy themselves, as opposed to another group who may harbour elaborate plans, carefully preparing a will, and so forth [15].

 The present study uses a phenomenological distinction between ego-syntonic and ego-dystonic suicidal ideation based on previous studies in our group. We have found that suicidal ideation “beyond one's own will” (i.e., ego-dystonic) was related to completed suicide in a severe depression with psychotic features for men and bipolar disorder for both men and women, while the more rational/wanted items on Beck's SSI were not related [16]. Previous studies have also shown that there were generally similar rates of adequate antidepressant treatment and also improvement in suicides and controls in the long-term course [17] and at suicide [18]. Consequently, the increased suicide risk for patients who report suicidal ideation “beyond one's own will” may be resistance to antidepressant therapy.

The aim of the present study was to compare ego-dystonic and ego-syntonic ideation during adequate antidepressant treatment, to identify any differential responses to treatment. The following questions were addressed: firstly, was ego-dystonic compared to ego-syntonic suicidal ideation more often reported during adequate treatment in the long-term course: secondly, had those who committed suicide during adequate antidepressant treatment more often reported ego-dystonic suicidal ideation earlier in their lives, indicating a lack of response to treatment for this type of ideation? Men and women were compared.

## 2. Materials and Methods

### 2.1. Sample

In the 1950s and 1960s, all in-patients at the Department of Psychiatry, University Hospital, Lund were rated on a multiaxial diagnostic schedule at discharge [19]. This database enabled the selection of patients with a prospectively rated severe depression/melancholia for an investigation into suicide.

**Figure 1:**
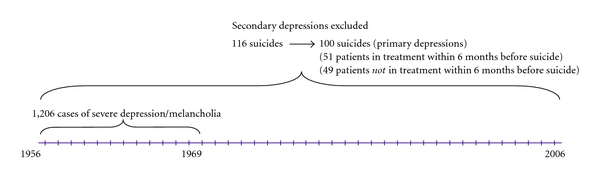
Flow-diagram for the sample of patients with severe depression admitted to the Department of Psychiatry, Lund University Hospital.

 A total of 1,206 patients received this diagnosis (506 men and 700 women). Their mortality was followed-up in three sessions—to January 1, 1984 to January 1, 1998, and to Feb 15 2010. There were 116 suicide victims up to 2010. Deceased persons were grouped according to the primary cause of death as classified by the Central Bureau of Statistics using the International Classification of Disease [20–22]. The sampling procedure is presented in Figure 1.

The case records were prepared for a thorough blind evaluation by omitting the last sheet with information on the suicide, as has previously been described [23], and a similar procedure was used at second and third follow-ups. Secondary depressions, mainly alcoholism, were excluded. This left us with 100 completed suicides, 44 men and 56 women, with a primary severe depression. Matched controls, one for each suicide, were selected from the total sample by the criteria of diagnosis, gender, year of birth, and index admission year, which meant that they were the same age on admission. The controls were selected on the basis of being alive and monitored up until the death of the suicide victim they matched and so were monitored for a similar length of time. 

A retrospective diagnosis according to DSM-IV [24] has been performed based on the symptoms reported in the records. It turned out that at least 91% of the patients met the criteria for a major depressive disorder with melancholic or psychotic features; of these, 47% were melancholic and 63% psychotic, and 22% had a bipolar disorder. The rates in suicides and controls were the same. 

Comorbid obsessional-compulsive symptoms blindly scored in the case records in the first follow-up did not significantly differentiate between suicides and controls (13% versus 8%). Anxiety prospectively scored on the multiaxial ratings did not differentiate (35% versus 33%). Being married at index admission (blindly rated) was significantly more common in female controls but social class did not differentiate for men or women [23]. Prospective scores (multiaxial ratings) of brittle/sensitive personality and heredity for psychosis were significantly more common in male suicides than male controls [23].

### 2.2. Suicidal Ideation

Suicidal ideation was scored based on the reports in the case records according to Beck's Scale for Suicidal Ideation—SSI [25], as described in a previous paper [16]. However, some types of ideation reported in the records did not fit in the basically rational thinking in SSI and those items were scored separately. Of the latter “suicidal ideation beyond one's own will” was significantly related to suicide.

The occurrence of ego-dystonic suicidal ideation, or “beyond one's own will” [16], was compared with ego-syntonic thoughts of suicide during adequate antidepressant therapy. These correspond to the dichotomy suggested by Ringel [14] and Akiskal [15]. The ratings were as follows.

- *Ego-dystonic suicidal ideation.* Unwanted urges to take one's own life; suicidal ideation “beyond one's own will”; fear of killing oneself; a compulsion to kill oneself; and nightmares about suicide. (Three cases of imperative hallucinations demanding suicide were found in the control group only; they overlapped with irrational/unwanted ideation in all but one case and were excluded.)
- *Ego-syntonic suicidal ideation* (items on Beck's SSI, English version [25]). Evidence of desire to take one's own life: item 4, desire to make an active suicide attempt; item 12, method considered; item 16, partial or complete preparations; item 17, suicide notes completed, and item 18, act in anticipation of death.

All scoring was performed by the same rater, who was blind to suicidal outcome and to the scoring of antidepressant therapy and repeated on a later occasion to enhance the reliability. (Beck's SSI was used for the scoring as well as items describing “suicidal ideation beyond one's own will” or ego-dystonic ideation [16].)

### 2.3. Antidepressant Therapy

Antidepressant therapy during the course of depression was evaluated according to Ottosson [26] for all follow-ups (1984, 1998, 2010) and was as follows.

1. Adequate doses were scored as follows: a full dose of at least 150 mg of tricyclic or tetracyclic antidepressants should be continued for at least 2 months. (A few patients who had received monoamine oxidase inhibitors (MAO) inhibitors were included.)
2. ECT (adequate treatment): a series of at least six treatments was considered adequate (all patients were given three treatments a week). (According to the tradition of the clinic, treatment was sometimes discontinued after 4 seizures. These cases were included if there was a persistent effect on depression.)
3. ECT and continuation treatment with antidepressants: continuation treatment should be initiated within 2 weeks after the last ECT. No sign of relapse of depression should have been reported.
4. Lithium-prophylaxis was also scored (and in one case valproatic acid).

Treatment with neuroleptics was also noted. Adequate treatment of depressive episodes was similar throughout the course of depression in suicides and controls and so was improvement, as described in a previous paper [17]. Suicidal ideation was scored as to whether it was reported during ongoing adequate antidepressant therapy or when untreated. Antidepressant medication should have been used at least four weeks in order to be considered to have an antidepressant effect, and a full course of ECT should have been completed. For those who received adequate antidepressant therapy, occurrence of ego-dystonic and ego-syntonic suicidal ideation during adequate treatment was scored, in order to evaluate whether relief of depressive symptoms also included relief of suicidal ideation.

 Finally, treatment at suicide was related to type of ideation previously reported. In all, 51 members of the sample were in contact with the department within six months before suicide (22 men and 29 women). This contact was supposed to concern their last episode.

The study was approved by Lund University Medical Ethics Committee 1985 and 2003.

### 2.4. Statistics

An *χ* ^2^ test was used for comparisons between groups and Fisher's exact test, when applicable. Wilcoxon's test was used for a comparison of ranks. A sign test was used for a comparison between type of suicidal ideation during adequate treatment, as almost all patients had received adequate treatment on some occasion and could therefore report suicidal ideation during treatment. By using a sign test, those who had reported both ego-dystonic and ego-syntonic ideation on different occasions were ascribed a zero value, and those who had reported only one type of ideation were compared.

## 3. Results

### 3.1. Report of Suicidal Ideation and Long-Term Treatment

Treatment of depressive episodes in the long-term course was compared for suicides and controls. A vast majority had received adequate antidepressant therapy at some time, 93% of the suicide group and 95% of the control group. Type of treatment, such as ECT, antidepressant pharmacotherapy, or mood-stabilisers did not vary. 

 Suicidal ideation could *not *be shown to occur more often among those who were less adequately treated. Within the suicide group, as well as the control group, suicidal ideators showed similar rates of adequately treated episodes as did nonideators (66% versus 79% in the suicide group and 76% versus 69% in the control group). Rates of ECT, antidepressant pharmacotherapy, and Lithium were similar for ideators and nonideators too.

### 3.2. Type of Ideation Related to Treatment and Long-Term Course

Suicides and controls showed similar rates of treatment and were analysed together. A sign test showed a significant increased risk for patients with ego-dystonic suicidal ideation as compared to ego-syntonic ideation during adequate antidepressant therapy (24 ego-dystonic only versus 8 ego-syntonic only; *P* < .004). 

**Figure 2:**
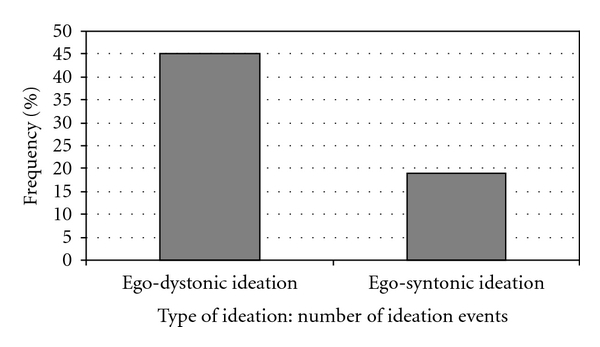
The proportion of reported ego-dystonic and ego-syntonic suicidal ideation during adequate treatment related to the total number of reported ego-dystonic and ego-syntonic ideation (percent).

**Figure 3:**
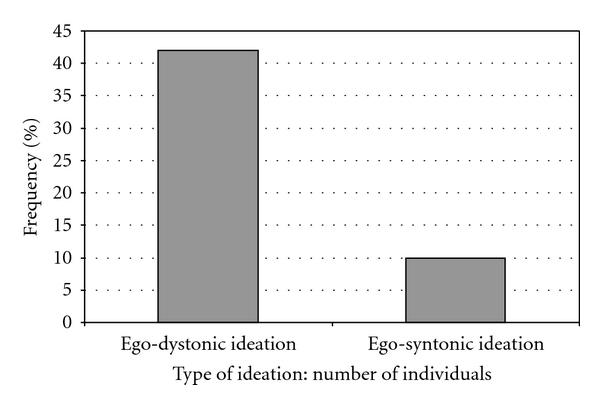
The proportion of individuals who reported ego-dystonic and ego-syntonic suicidal ideation during adequate treatment only, related to the total number of individuals who had at some time reported ego-dystonic and ego-syntonic ideation (percent).

The proportion of ego-dystonic and ego-syntonic ideation events reported during adequate treatment in relation to the total number of ideation events is presented in Figure 2. The proportion of individuals who reported ego-dystonic or ego-syntonic ideation during antidepressant therapy only in relation to the total number of individuals who reported the type of ideation is presented in Figure 3. A clear peak for ego-dystonic compared to ego-syntonic suicidal ideation was found during adequate treatment.

### 3.3. Type of Ideation Related to Treatment at Suicide

Suicidal ideation previously reported was compared for those patients who had been in contact and adequately treated before suicide and those who were untreated, being in contact or not (Table 1). Males who committed suicide during adequate treatment had more often previously reported ego-dystonic suicidal ideation in comparison to all untreated (7/15 versus 5/29, chi-square = 4.32, *P* < .038). Furthermore, if only those who were in contact before suicide were compared, a significant difference for ego-dystonic ideation between treated and untreated remained (7/15 versus 0/7, Fisher's exact test *P* = .038). All the seven patients with ego-dystonic ideation who committed suicide despite adequate treatment had previously been diagnosed with psychotic features. Of these, two were prescribed additional neuroleptics, only one as an antipsychotic. There was a nonsignificant trend for males to report ego-dystonic suicidal ideation during treatment more often than women (7/15 versus 3/17, chi-square = 3.12; *P* = .078).

## 4. Discussion

Firstly, ego-dystonic suicidal ideation was shown to be more commonly reported during adequate antidepressant therapy as compared with ego-syntonic suicidal ideation. Secondly, the ego-dystonic ideation previously in life was more often reported by men who committed suicide during antidepressant treatment as compared to other male suicides, being in contact at suicide or not. We do not know whether the patients who previously had reported ego-dystonic suicidal ideation also had these thoughts at the time of suicide. However, the fact that ego-dystonic ideation also appeared resistant to antidepressant treatment in the long-term course justifies an assumption that suicide may have been preceded by this type of suicidal thought. The association with accomplished suicide was found only in the male group. It is possible that men more often act on this type of suicidal feeling, as men are more prone to fatal suicidal acts [27]. The occurrence of ego-dystonic suicidal ideation and accomplished suicide during adequate antidepressant therapy may be due to a lack of response for this type of suicidal ideation. In a previous study, we found evidence that urges to make suicide attempts may remain during adequate treatment with antidepressants, though the acts were often interrupted [17]. That finding is compatible with the occurrence of ego-dystonic suicidal ideation during antidepressant therapy found in the present study. Ego-syntonic suicidal ideation appears to show a better response to antidepressant therapy as compared to ego-dystonic suicidal ideation. Perhaps ego-syntonic ideation is part of a depressive disorder and consequently reduced when there is a relief of depression. On the other hand, ego-dystonic ideation appears to show a poor response, indicating that it may occur independent of depressive symptoms. In another previous study [16], we found a correlation between ego-dystonic suicidal ideation (“beyond one's own will”) and psychosis. All the men who committed suicide during antidepressant therapy had suffered depression with psychotic features. As ego-dystonic suicidal ideation both appeared resistant to antidepressant treatment and was also related to psychosis, there is an interesting possibility that this type of suicidal ideation may actually be psychotic in nature. According to other investigators, patients with psychotic depression seem to be much more likely to exhibit high levels of suicidal ideation following treatment compared with patients without psychotic features [28], which is compatible with the present findings. Risperidone augmentation to antidepressants has also recently been shown to reduce suicidal ideation in major depressive disorder [29], a finding compatible with a hypothesis of “psychotic suicidal ideation”. However, none of these studies considered the type of ideation.

The present study deals with a sample of severely depressed patients. Severe depression has been shown to be a predominant diagnosis among suicide victims [30] and to have an increased risk for completed suicide as compared to major depression without severe features [31, 32]. The records were well written and very informative and a great number of statements on suicidal ideation were available, but an important weakness of this study is the lack of systematic inquiry of suicidal ideation. On the other hand, the study builds on continuous reports in the case records noted at the time, thereby minimising the effect of recall bias, which is a strength. Furthermore, in making a comparison between the occurrences of different types of ideation during treatment, it is assumed that patients are equally prone to communicate those types of suicidal ideation, whether under treatment or not. This is probable but cannot be proved. There were some differences between suicides and controls concerning personality and marital status that is shown in a previous paper [23]. However, there is no indication these would influence the different responses for types of ideation, and the improvement rates on antidepressant therapy were the same for suicides and controls. Type of suicidal ideation was compared during different types of treatment, which may have different modes of action. However, the common objective is relief of depression through the use of ECT, antidepressants, and mood-stabilisers, which work in much the same way. Finally, the antidepressants prescribed were generally tricyclics, which are no longer a first choice. However, a similar effect of these drugs as compared to selective serotonin reuptake inhibitors and serotonin norepinephrine reuptake inhibitors (SSRI and SNRI) on suicidal behaviour has been proposed [32, 33] and also generally a better effect on severe depression [34, 35].

## 5. Conclusion

Ego-dystonic suicidal ideation appeared to be more frequent during adequate antidepressant therapy than ego-syntonic suicidal ideation. The distinction between these types of ideation during antidepressant treatment therefore appears to be important in efforts to predict and prevent suicide. We propose that ego-syntonic ideation may be a part of a depressive disorder and thereby reduced by antidepressant therapy, while ego-dystonic ideation occurs more frequently during adequate therapy, perhaps indicating that it may be psychotic in nature.

## Figures and Tables

**Table 1:** Ego-dystonic and ego-syntonic suicidal ideation related to treatment by contact and gender.

	Men *N* = 44			Women *N* = 56		
	Ego-dystonic ideation	Ego-syntonic ideation	Men total	Ego-dystonic ideation	Ego-syntonic ideation	Women total
Contact and treatment within 6 months before suicide	7	11	15	3	13	17
Contact and *no* treatment within 6 months before suicide	0	6	7	6	10	12
No contact before suicide	5	17	22	3	15	27
Ideation Total	12	34	44	12	38	56

(Note that one patient could have both or none of the types of ideation. Thus the rows and columns could not be totalled.)

Ego-dystonic ideation reported by men in contact and treated versus all other men: chi-square = 4.31, *P* < .038.

Ego-dystonic ideation reported by men in contact and treated versus in contact and untreated: Fisher's exact test = 0.038.

Ego-dystonic ideation in men versus women in contact and treated: chi-square = 3.12, *P* < .077.
